# Robust tunable plasmon induced transparency in coupled-resonance finite array of metasurface nanostructure

**DOI:** 10.1038/s41598-020-78795-0

**Published:** 2021-01-13

**Authors:** Jie-Tao Liu, Zhi Liu

**Affiliations:** 1 School of Physics and Optoelectronic Engineering, Xidian University, Xi’an, 710071 China; 2 Xi’an Key Laboratory of Computational Imaging, Xi’an, 710071 China; 3 State Key Laboratory on Integrated Optoelectronics, Institute of Semiconductors, Chinese Academy of Sciences, Beijing, 100083 China

**Keywords:** Nanophotonics and plasmonics, Sub-wavelength optics

## Abstract

Robust and dynamically polarization-controlled tunable plasmon induced transparency (PIT) resonance in designed finite-array nanostructures metasurface is demonstrated, where sharp resonance is guaranteed by design and protected against large geometrical imperfections even for micro-zone sub-array. By employing the explicit analysis of near-field characteristic in the reciprocal-space based on the momentum matching, and the far-field radiation features with point-scattering approach in real-space sparked from Huygens’s principles, the physics of interference resonance for plane-wave optical transmission and reflection of the metasurface is theoretically and thoroughly investigated. The distinctive polarization-selective and Q-tunable PIT shows robust features to performance degradations in traditional PIT system caused by inadvertent fabrication flaws or geometry asymmetry-variations, which paves way for the development of reconfigurable and flexible metasurface and, additionally, opens new avenues in robust and multifunctional controllable nanophotonics device design and applications.

## Introduction

Plasmon-induced-transparency (PIT) is a widespread wave scattering phenomenon associated with a peculiar coupled interference effect. Since its first demonstration^1^ in the plasmonic nanostructures, PIT has been widely and thoroughly studied both theoretically and experimentally^2–4^ due to a large variety of pivotal and potential applications in sensing, filter, switches, slow-light, and nonlinearity. However, the study of PIT with reconfigurable dynamic tunability poses several challenges. Many works reported tunable PIT resonance by employing the geometrical asymmetry/variations, which is inherently static and the control of channels for the PIT windows are rarely studied^3^. Favorably, phase-change-materials, graphene, magnetic-materials, and nonlinear dielectric nanostructures have auspiciously emerged as a promising alternative and platform for dynamic control of PIT, and are expected to complement or even replace plasmonic nanostructures for a wide range of potential applications^5^.

Recently, carefully designed plasmonic nano-antennas taking advantages of the polarization-selective excitation and evolution of the split-ring-resonator like unit have been proposed to shed light on dynamic PIT construction, not only providing a notable sequential polarization-controlled switchable PIT channels, but also flexibility and configurability^3^. Nevertheless, these devices perform structure-parameters sensitivity, which conversely challenges the state-of-art of fabrication and owns inferior tolerance to fabrication errors and flaws, leading to deficiency of robustness of the PIT resonance for practical applications. Tunable PIT with dynamic manipulation properties is unresolved for PIT with immunity against fabrication imperfections/flaws and geometrical variations. Topological optics is inspiring a new perspective on design of nanophotonics metasurface and devices^6,7^. The manipulation of photonics by employing nanoscale photonics structures with exotic properties has been well demonstrated with fascinating designs^6–8^. Due to their simple construction and efficient tunability, metasurface using nanoslits or slot antennas topology as building blocks has been identified as a versatile platform to study topological nature and manipulation of photonics for helicity dependent directional surface plasmon polaritons (SPP) excitation^9^, flexible control of in-plane SPP shaping and focusing or far-field radiation^10,11^, chirality of asymmetric transmission^12^, parity-time symmetry breaking^13^, and spin optics in metasurface^14^.

In this study, we introduce a class of compact slits nano-antennas array in metallic film that lift all these limitations and show their feasibility to be dynamically controlled by incidence polarization, where robust PIT with tunable quality factors is demonstrated. The physics of the generation, evolution, and annihilation of the PIT, and the immunity of the proposed system to the asymmetry geometry parameters variation and micro-zone-array are investigated and illustrated by exploring the dispersion characteristics in the real-space and the reciprocal-space. The simulated results are verified by the point-scattering approach model theoretical predictions, where the interference of the field in the slits and the far-field radiation features are revealed. The results pave the way for reconfigurable, flexible multifunctional metasurface and robust nanophotonics device design and applications.

### Proposed metasurface and theoretical calculation method

**Figure 1:**
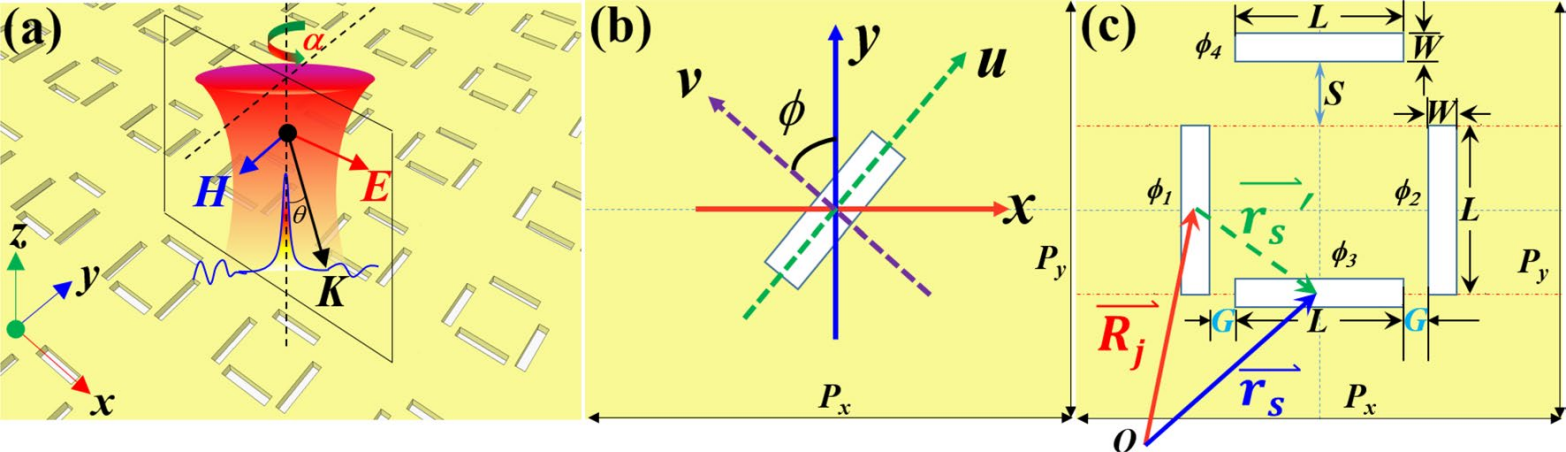
**(a)** Schematic of the proposed nanostructures with particular identical rectangular slits. **(b)** Scheme of the unit cell showing one slit and corresponding coordinates. **(c)** Coordinate vector and unit cell of the proposed metasurface.

The proposed topology consists of periodic rectangular nanoslits antenna array in gold film on substrate. Figure 1a illustrates the schematic of plasmonic nanostructures. A 50-nm-thick gold film was deposited on a glass substrate. The incidence is denoted as linear polarized light with an angle of polarization (AOP) α. Figure 1b shows the scheme of the unit cell slit’s orientation and the corresponding coordinate system. The unit cell with perforated specifically arranged resemble slot antennas (*L* = 200 nm, *W* = 40 nm) is shown in Fig. 1c. The periods *P*_*x*_, *P*_*y*_ are set as 600 nm equally along both *x* and *y* directions.

The geometrical parameters shift *S* and gap *G* are denoted in Fig. 1c, respectively. The rotation coordinate system denotes as *u–v* and the translational coordinate system as *x–y* are shown in the Fig. 1b. The coordinate vector system for the proposed metasurface is shown in Fig. 1c. For the case of a rectangular-shaped nano-slot in metal film with a high aspect ratio, the resonance and amount of SPP generation are significantly dependent on the orientation of the incident polarization^15–18^. Different from the metallic nanobars where the plasmonic resonance mode is excited with polarization along the long-arm, the SPP can only be excited when the polarization direction of the incident light is perpendicular to the metal slits.

The physics and model of optical transmission of periodic subwavelength holes array (SHA) nanostructures have been vastly and thoroughly studied, where several model such as the coupled-mode equation^19^, hybrid-wave model^20^, and the ab initio theory of Fano-formula^21^ were proposed. Meanwhile, the resonance role of SPP modes was studied by reciprocal-space momentum-match conditions. For single-interface SPP, the dispersion relation can be described as:

1 $$K = k_{spp} = 2\pi \sqrt {\varepsilon_{m} \varepsilon_{d} /(\varepsilon_{m} + \varepsilon_{d} )} /\lambda ,$$

where *ε*_*d*_ and *ε*_*m*_ are the relative permittivity of the dielectric and the metal. The dispersion relation of the SPP Bloch mode generated under the Bragg coupling condition can be obtained by the momentum match:

2 $$\mathop{{k}_{spp}}\limits^{\rightharpoonup} = \mathop{{k}_{in}}\limits^{\rightharpoonup \prime \prime } + i\frac{2\pi }{{P_{x} }}\mathop{{u} _{x}}\limits^{\rightharpoonup} + j\frac{2\pi }{{P_{y} }}\mathop{{u}_{y}}\limits^{\rightharpoonup}$$

where $\mathop{{k} _{in}}\limits^{\rightharpoonup \prime \prime }$ is the component of wave vector ***k*** parallel to the array surface, $\mathop{{u}_{x}}\limits^{\rightharpoonup}$ and $\mathop{{u} _{y}}\limits^{\rightharpoonup}$ are the unit vector in plane. Instead of straightforward description of the transmission, the reciprocal-space method relies on a priori definition of resonance as unperturbed smooth surface mode without the nanostructures. In 2005, Genet et al. presented a real-space Huygens description instead of the more conventional reciprocal-space description of interfering resonance features in the SHA^22^. However, the anisotropic designed holes arrays increasingly utilized in the metasurface for flexible control and modulation of electromagnetic wave are seldom treated. Recently, the SPP far-field radiation in an anisotropic SHA metasurface is demonstrated^11,23^. In the point-scattering model, the unit cell of the hole openings are treated as resonance point-scatter. In Fig. 1b, the angle *ϕ* between the *v* axis (also referred to as the normal direction of a nanoslit) and *y* axis is defined as the rotating angle of the nanoslit. By setting the first, lower-left nanoslit’s center as the origin ***O*** of the whole system, the contribution of all the point-scatter to the transmission spectra can be processed based on the Huygens’ Principles.

The disposition vector of the center of the first nanoslit in the *j*th (the *m*th row and the *n*th column) supercell is $$\mathop{{R}_{j}}\limits^{\rightharpoonup} = mP_{x} \mathop{x}\limits^{\rightharpoonup} + nP_{y} \mathop{y}\limits^{\rightharpoonup}$$. The unit vector for the position vector is $$\widehat{u}_{j} { = }\overset{\lower0.5em\hbox{$\smash{\scriptscriptstyle\rightharpoonup}$}}{{\text{R}}}_{j} /\left| {\overset{\lower0.5em\hbox{$\smash{\scriptscriptstyle\rightharpoonup}$}}{{\text{R}}}_{j} } \right|$$. In one unit cell, the relative coordinate of the center of the *j*th nanoslit’s center relative to the first nanoslit’s center in the same supercell is set as $$\mathop{{r}_{s}}\limits^{\rightharpoonup \prime}$$ (Fig. 1c). The absolute coordinate of the *j*th nanoslit’s center can be obtained as $$\mathop{{r} _{s}}\limits^{\rightharpoonup} = \mathop{{R} _{j}}\limits^{\rightharpoonup} + \mathop{{r} _{s}}\limits^{\rightharpoonup \prime}$$.

With the propagation direction of SPP expressed as $\widehat{u}_{spp} = (\widehat{v} \cdot \mathop{{E}_{in}}\limits^{\rightharpoonup} ) \cdot \widehat{v}$, where the polarization of the incident plane wave is set as the direction along the *y*-axis ($\mathop{{E}_{in} }\limits^{\rightharpoonup} = \mathop{y}\limits^{\rightharpoonup}$) for TE (Transverse Electric), *x*-axis ($\mathop{{E} _{in}}\limits^{\rightharpoonup} = \mathop{x}\limits^{\rightharpoonup}$) for TM (Transverse Magnetism), and the excited SPP propagation vector of the nanoslits $\mathop{v}\limits^{\rightharpoonup} = \mathop{y}\limits^{\rightharpoonup} \cos \phi - \mathop{x}\limits^{\rightharpoonup} \sin \phi .$ The full polarization behavior of the SPP is contained in the tensorial $\widehat{u}_{j} \otimes \widehat{u}_{j}$ nature of the elementary scattering matrix. Assuming that the scattering matrix of the far-field is spherical so that the intensity of the transmission for the metasurface can be calculated^11,22,23^,

3 $$Ratio = \frac{1}{{T_{0} }}\left| {t_{scatt} \cdot \widehat{u}_{spp} } \right|^{2} = \frac{1}{{T_{0} }}\left| {\sum\limits_{{\{ r_{s} \ne {0}\} }}^{{}} \begin{gathered} s(|K|)\exp ( - i\frac{\pi }{4})\left[ {\frac{{{\text{Re}} (|K|)}}{2\pi }} \right]^{1/2} \cdot \hfill \\ \frac{{\exp (i|K||\overset{\lower0.5em\hbox{$\smash{\scriptscriptstyle\rightharpoonup}$}}{{r_{s} }} |)}}{{\sqrt {|\overset{\lower0.5em\hbox{$\smash{\scriptscriptstyle\rightharpoonup}$}}{{r_{s} }} |} }}\exp (i\overset{\lower0.5em\hbox{$\smash{\scriptscriptstyle\rightharpoonup}$}}{{k_{in} }} \cdot \overset{\lower0.5em\hbox{$\smash{\scriptscriptstyle\rightharpoonup}$}}{{r_{s} }} )\widehat{u}_{j} \otimes \widehat{u}_{j} \cdot \widehat{u}_{spp} \hfill \\ \end{gathered} } \right|^{2}$$

where the spectrum for *m *= 1, *n* = 1 are defined as a normalized coefficient *T*_0_. Under point-scattering limit, the shape factor *S* (*|K|*) can be replaced with a constant. This relates the present formulation to the two-dimensional Green function of the surface-wave with no need to investigate the full three-dimensional Green function half-space problem^22^. For a given hole, the incoming field is converted into two-dimensional surface waves that propagate away from the hole as a spherical (Huygens) wave. Based on the point-scattering-approach, the plane-wave optical transmission of the designed metasurface for arbitrary angles and polarizations can be evaluated and predicated theoretically.

**Figure 2:**
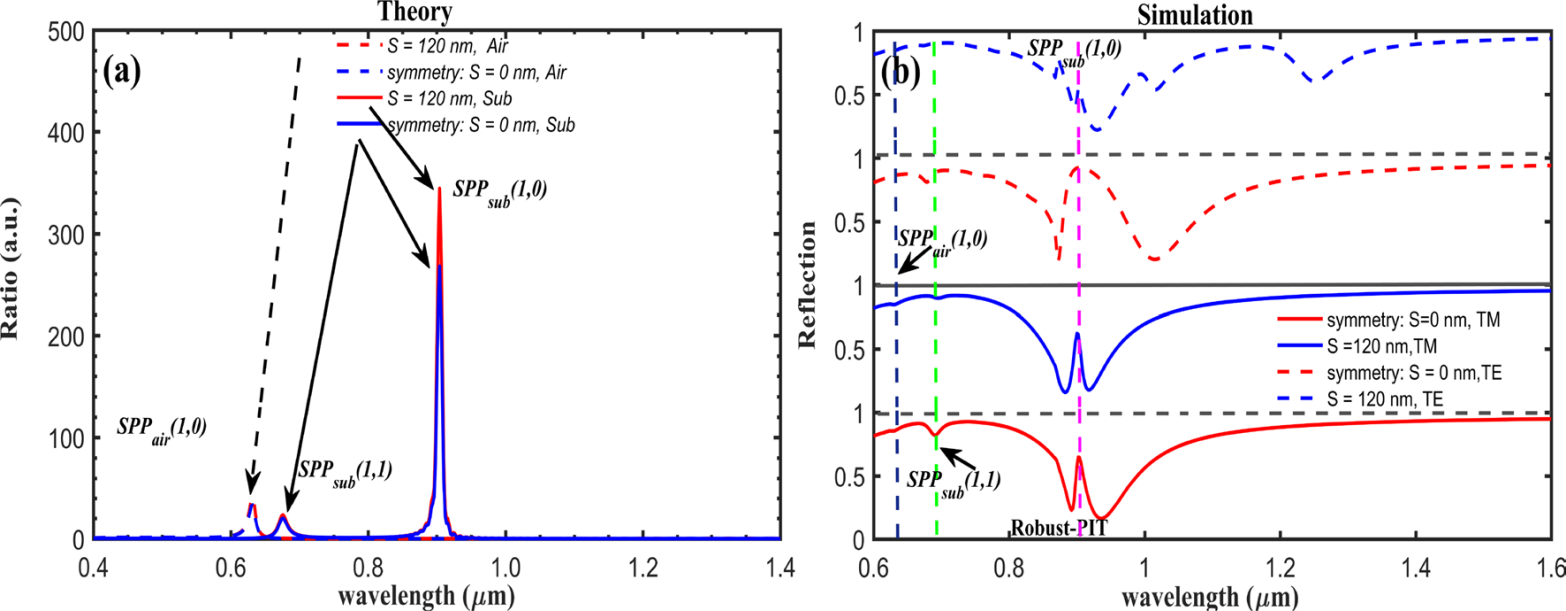
**(a)** Theoretic model calculated transmission coefficient at TM (solid-lines)/TE (dashed-lines) incidence for 100 × 100 periodic unit, and **(b)** numerical simulated reflection spectra of the metasurface. “Sub” and “Air” indicate the substrate and upper air interfaces.

The intensity of transmission spectra for both TM/TE plane-wave at normal incident for the symmetric/asymmetric structure are theoretically calculated using Eq. (3) for convergent values at *m* *=* *n* = 100, and the results are shown in the Fig. 2a. It is demonstrated that the PIT resonances are directly linked to the spectral position of the Au/glass interface SPP Bloch Mode, which shows robust polarization-insensitive features. As studied and shown by Pacifici et al., the positions by the point-scattering model theory prediction should be the transmission minimum^23^. To verify the theory prediction, the full-field electromagnetic calculations were performed using the finite difference time domain (FDTD) method. The permittivity of Au was taken from the experiment data^24^, while the refractive index of the glass substrate was fixed at 1.45. The calculated reflectance spectra are shown in the Fig. 2b. Clear transparency windows with narrow peaks are observed at 904 nm wavelength, for both the system with and without broken-symmetry. Excellent agreement between the theory and the simulation is observed.

## Discussions

### Optical response and tunability of the nanostructure

The PIT is maintained and obtained in the system without geometry symmetry-breaking, which shows extraordinary immunity to the symmetry-breaking employed and usually required in the traditional plasmonic nanostructure using the resemble resonance nanobar/nanoslit unit. The in-plane interference of the surface wave by the scattering of light from the arranged nanoslits is attributed to the variation of the reflection spectra for different gaps of the nanoslits.

**Figure 3:**
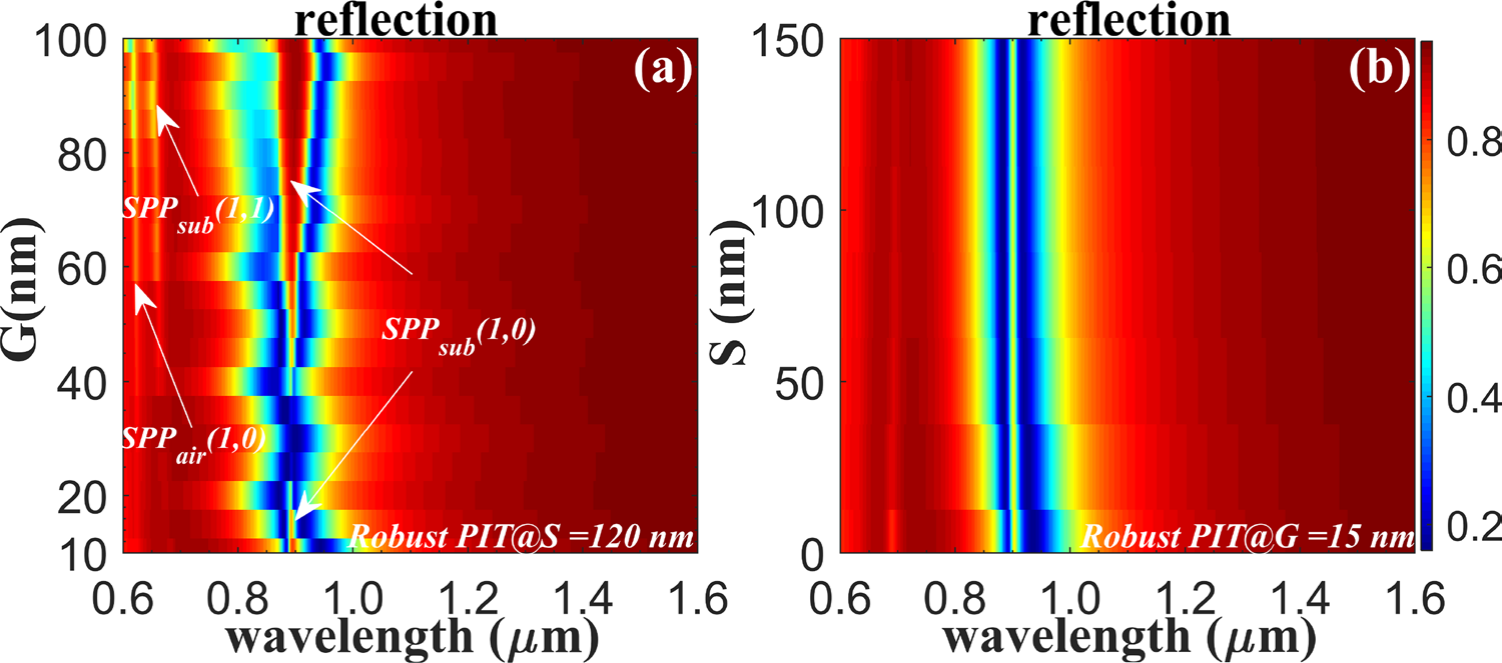
Calculated reflection spectrum as a function of varied Gaps values *G* **(a)**, and shift *S* **(b)** for *TM*-polarization. Robust PIT spectra immune to marginal geometry variations is observed.

The results in the Fig. 3a show clear emerging and vanishing of the PIT peak for different gap values, where the PIT peak is shrinking and fading for increased gaps below 20 nm, while the notable regeneration of the PIT with broaden and enlarged peak is observed for further increased gaps. The result shows quite unique trends featured with unusual fading and revival of the PIT resonance when increasing the gaps *G*. Hence, once the distance for varied coupling is increased or decreased, the PIT resonances position moves. The oscillatory behavior as a function of varied gaps is also observed as theoretically predicted from the surface-wave mediated interference model, where the first-order interference between SPP and these out-of-plane component at the subwavelength scattering sites formed by the slits openings gives rise to the observed spectra^23^. The PIT-like resonance is maintained for vast range of gaps, which shows relatively robust performance to fabrication flaws or imperfection. To further verify this, the rotation angles (*ϕ*_1_, *ϕ*_2_, *ϕ*_3_, *ϕ*_4_) of the slits are slightly and randomly changed, where by theory prediction, besides of the shrink ratio values, the unchanged fixed peak position centered at 904 nm is still observed agreeing with the simulation results.

At the same time, changing the distance (Shift *S*) between the nanoslits vertical to the incidence polarization will not significantly affect the position of the PIT resonance due to the fact that the slot-antenna’s polarization-excitation-selective property. Figure 3b shows the reflectance spectrum at normal incidence as a function of varied shifts of the nanoslit 1 (see Fig. 1c), where the PIT resonance effect shows slight changes for shifts below 40 nm, and the transparency window shows negligible and almost unchanged features for increased shifts. By carefully designing utilizing the polarization-selective bound-charge-oscillator like slot antenna unit^15,17^, the singular features of the PIT resonance in the system is reported for the first time, to the best of our knowledge, that the topologically robust PIT resonance is demonstrated in metasurface for both symmetric and asymmetric unit. Most works indicated that asymmetry was crucial to realize PIT when the interaction of dipole resonance and the plasmon resonance modes in the nanostructure is considered. Here we propose structure that is not limited by the asymmetry condition and PIT resonance with robustness features shows immunity to the asymmetry geometry variation.

**Figure 4:**
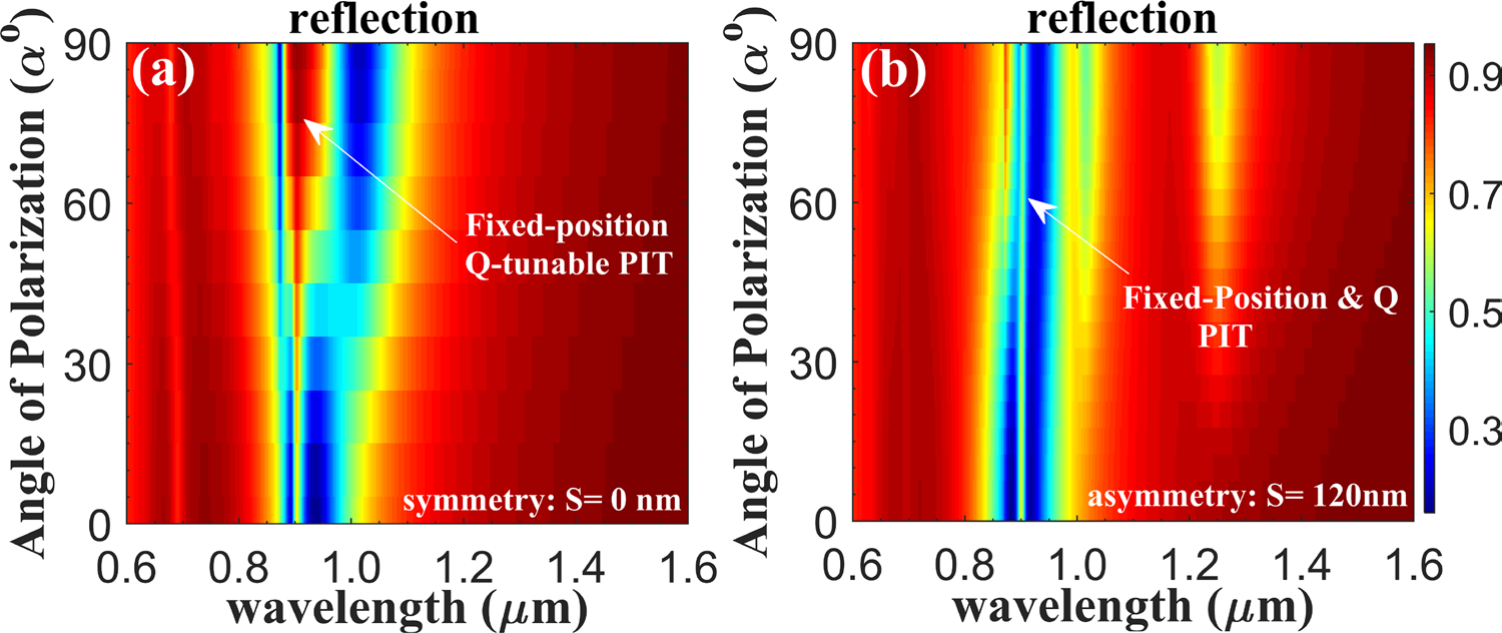
**(a)** Reflection spectra as a function of the polarization orientation angles for symmetric (*G* = 15 nm, *S* = 0 nm) structure. **(b)** Reflection spectra as a function of polarization orientation angles for asymmetric (*G* = 15 nm, *S* = 120 nm) geometry. The PIT-like spectra is robust centered at fixed resonance position.

For varied incidence polarization orientation, the tunability of the system with fascinating sequentially switchable PIT resonance peaks with tuned quality factor (Q-factor) and contrast is obtained, where dynamic PIT with tunable quality can be efficiently tailored by adjusting the incidence polarization. The result in Fig. 4a exhibits clear evolution of the PIT resonance, where broaden resonance associated with almost fixed resonance position is demonstrated for increased angles of polarization. The resonance quality factors of the system with minimum 9 nm linewidth can be gradually tuned by controlling the AOP. For asymmetry structures, the PIT resonance is also observed from the results in Fig. 4b. Q-tunable robust PIT in designed metasurface immune to the geometry symmetry/asymmetry is obtained.

Furthermore, there has been plenty of designed controllable light beams with tailored wave-front and vector vortex are realized using coded metasurface or spatial light modulator (SLM), the fruitful dynamic PIT resonance with striking features inspired by manipulating the incidence polarization shows remarkable potential for dynamic metasurface devices design and applications.

### Dispersion manipulation and theoretical calculation of Bloch surface mode supported

**Figure 5:**
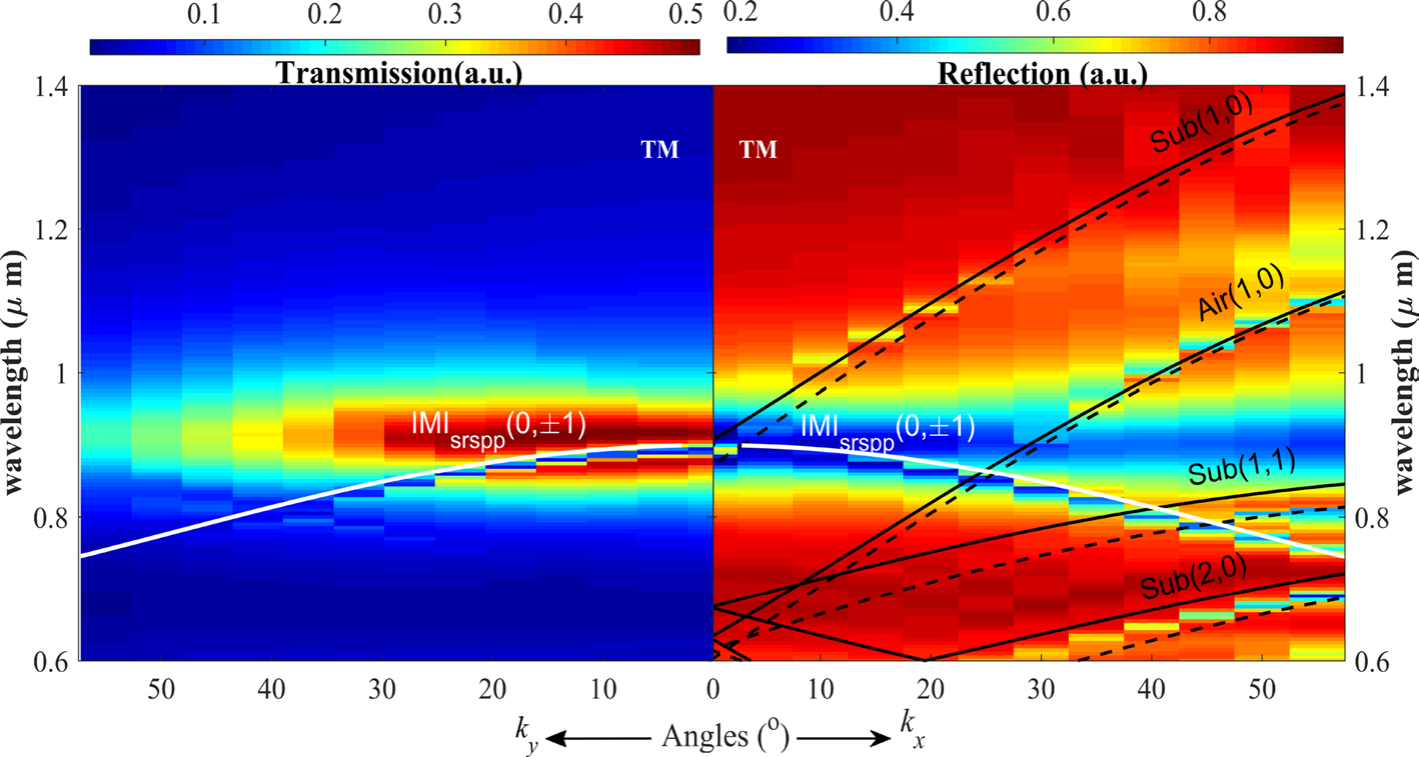
Calculated dispersion of the system for TM polarization incidence. Calculated transmission spectra dispersion (left panel). Right panel: Calculated reflection spectra dispersion. The superimposed white solid line denotes the calculated dispersion for asymmetry (air–Au–glass) three-layer (insulator–metal–insulator, IMI) waveguide-mode. The superimposed black lines are the dispersion curves of air and substrate SPP modes (solid lines) and wood anomaly (dashed lines). Robust PIT-like effect is observed.

**Figure 6:**
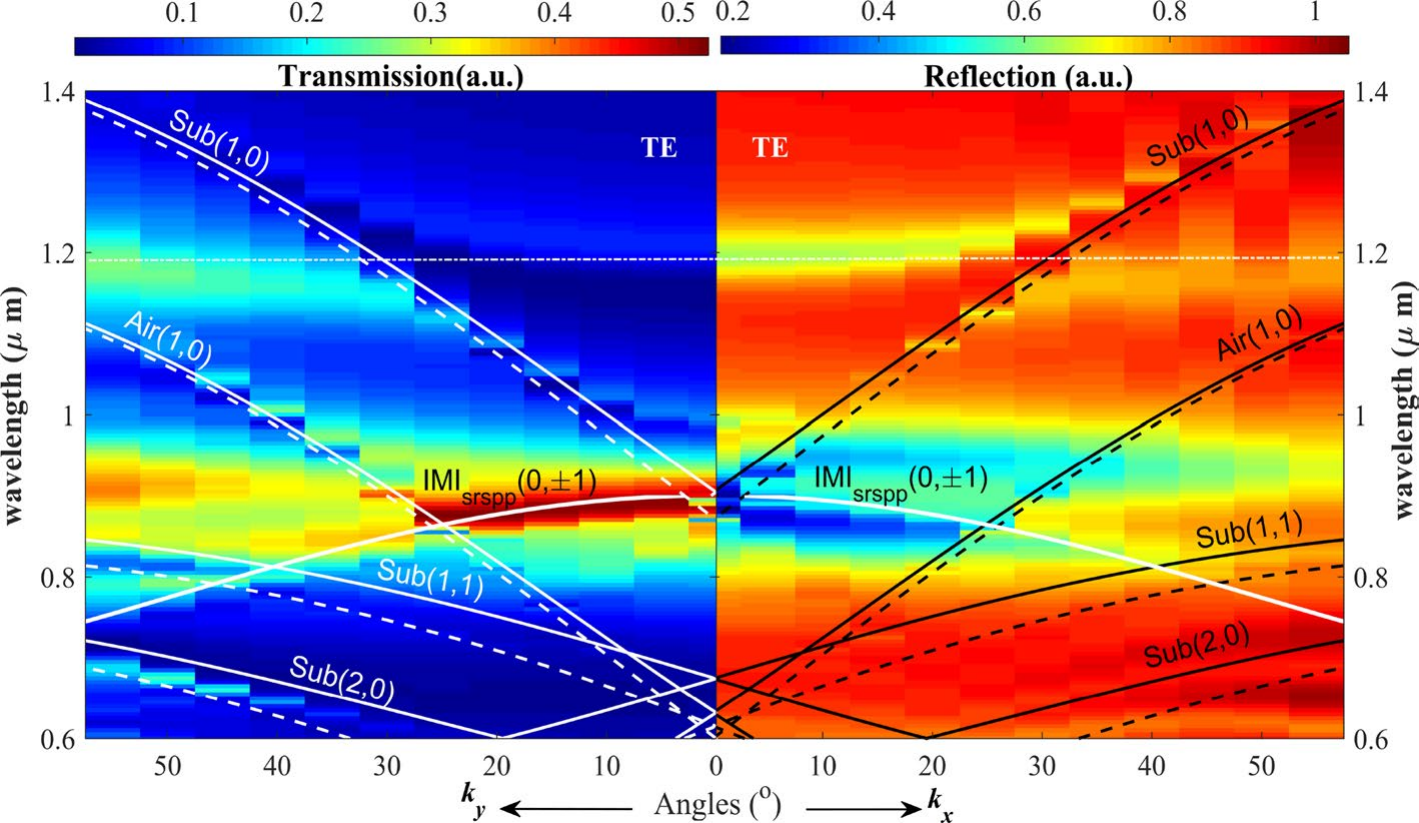
Calculated dispersion of the system for TE polarization incidence. Left panel: Calculated transmission spectra as a function of frequency and in-plane wave vector *k*_*y*_. Right panel: Calculated reflection spectra as a function of frequency and in-plane wave vector *k*_*x*_. The superimposed white solid line denotes the calculated dispersion curves for SPP Bloch-mode and the asymmetry IMI waveguide-mode. The superimposed dashed lines are the dispersion curves for wood anomaly. Robust PIT resonance is observed for large angles-variations.

To gain clear systematic properties of the system, the topological properties are investigated by exploring the dispersion of the nanostructure. The results in Figs. 5 and 6 show the dispersion calculated band for TM-polarization and TE-polarization for both *k*_*x*_, and *k*_*y*_ momentum using the reciprocal-space theory (Eq. 2).

The PIT-like resonance is revealed origins from the directional excitation coupled Bloch-surface-wave. It is crucial to dynamically control PIT resonances while maintain robust profile features. However, typically emerged PIT system introduces geometry asymmetry and consequently static tunability, hampering their use for dynamic, robust, practical applications. The active control of plasmonic PIT resonance in the proposed metasurface can be implemented by efficiently adjusting the incidence polarization. The result paves way for active engineering of robust PIT and dynamic nanophotonics devices. The compactness, the simple topology of the system, and the ease of engineering and tailoring of the resonance show potential and a wide class of promising design and applications for dynamic nanophotonics devices. The results provide a useful design strategy for plasmonic multifunctional nanostructures and versatile metasurface^25–29^.

## Conclusions

We demonstrate that the narrow PIT-like resonances can be guaranteed by carefully designed metasurface without stringent geometrical requirements, and with immunity to structural variations and misalignment. The PIT resonance is not disturbed by any coupling distance/asymmetry/polarizations; the PIT resonance can shift and is still sensitive to the angles of the incident light. Robust PIT with unique properties of versatile tunability can be achieved by adjusting the incident angles/polarization orientation, where high-Q PIT at different wavelengths and PIT at fixed position with tunable Q values can be achieved in one system. We envision that the result can offer new perspectives for the generation of multifaceted nanophotonics devices in many applicative fields, including robust sensing, filter, ultrafast switches or metasurface devices, by circumventing the performance degradations caused by fabrication flaws and maintain robust features without stringent geometrical requirements.
